# In Silico Study of Rett Syndrome Treatment-Related Genes, *MECP2*, *CDKL5*, and *FOXG1*, by Evolutionary Classification and Disordered Region Assessment

**DOI:** 10.3390/ijms20225593

**Published:** 2019-11-08

**Authors:** Muhamad Fahmi, Gen Yasui, Kaito Seki, Syouichi Katayama, Takako Kaneko-Kawano, Tetsuya Inazu, Yukihiko Kubota, Masahiro Ito

**Affiliations:** 1 Advanced Life Sciences Program, Graduate School of Life Sciences, Ritsumeikan University, Kusatsu, Shiga 525-8577, Japan; gr0343rp@ed.ritsumei.ac.jp (M.F.); sj0048hh@ed.ritsumei.ac.jp (G.Y.); sj0036kf@ed.ritsumei.ac.jp (K.S.); 2 Department of Pharmacy, College of Pharmaceutical Sciences, Ritsumeikan University, Kusatsu, Shiga 525-8577, Japan; s-kata@fc.ritsumei.ac.jp (S.K.); takanek@fc.ritsumei.ac.jp (T.K.-K.); tinazu@fc.ritsumei.ac.jp (T.I.); 3 Department of Bioinformatics, College of Life Sciences, Ritsumeikan University, Kusatsu, Shiga 525-8577, Japan; yukubota@fc.ritsumei.ac.jp

**Keywords:** Rett syndrome, intrinsically disordered region, phylogenetic profile analysis, post-transcriptional modification, methyl-CpG-binding protein 2, cyclin-dependent kinase-like 5, forkhead box protein G1

## Abstract

Rett syndrome (RTT), a neurodevelopmental disorder, is mainly caused by mutations in methyl CpG-binding protein 2 (*MECP2*), which has multiple functions such as binding to methylated DNA or interacting with a transcriptional co-repressor complex. It has been established that alterations in cyclin-dependent kinase-like 5 (*CDKL5*) or forkhead box protein G1 (*FOXG1*) correspond to distinct neurodevelopmental disorders, given that a series of studies have indicated that RTT is also caused by alterations in either one of these genes. We investigated the evolution and molecular features of MeCP2, CDKL5, and FOXG1 and their binding partners using phylogenetic profiling to gain a better understanding of their similarities. We also predicted the structural order–disorder propensity and assessed the evolutionary rates per site of MeCP2, CDKL5, and FOXG1 to investigate the relationships between disordered structure and other related properties with RTT. Here, we provide insight to the structural characteristics, evolution and interaction landscapes of those three proteins. We also uncovered the disordered structure properties and evolution of those proteins which may provide valuable information for the development of therapeutic strategies of RTT.

## 1. Introduction

Rett syndrome (RTT; OMIM entry #312750) is a rare disease that was first described by Andreas Rett in 1966 [1]. It is characterized by severe impairment such as deceleration of head growth, loss of speech, seizures, ataxia, movement disorder, and breathing disturbance [2]. Alterations in methyl CpG-binding protein (*MECP*)*2*, an X-linked gene involved in the regulation of RNA splicing and chromatin remodeling, were confirmed in approximately 95% of individuals diagnosed with RTT [3], while the others were confirmed in either cyclin-dependent kinase-like (*CDKL*)*5* or forkhead box protein (*FOXG*)*1* alterations as atypical cases of RTT [4,5]. The mutations in *MECP2* are generally paternally derived. Thus, this syndrome mainly affects girls, and the age of onset varies from 6 to 18 months [2,6]. Additionally, Rett syndrome can also affect males with severe phenotype and early lethality following the inactivation of the sole X-linked copy of *MECP2* [7]. In a rare case, it can also exist as somatic mosaicism or co-occur with Klinefelter syndrome in males [8,9]. Even though the causative genes have been determined, the infrequent clinical phenotypes yield to the difficulty in diagnosis. Further, diagnosis may be challenging as many of the clinical features overlap with those of other neurological and neurodevelopmental disorders, and mutation in *MECP2*, *FOXG1*, and *CDKL5* can also cause neurodevelopmental disorders distinct from RTT [10]. As a result, subsequent studies have suggested that alterations in either *CDKL5* or *FOXG1* should be classified as a distinct disorder from RTT as the majority of cases showed some differences in clinical features [11,12,13] Moreover, recent studies have suggested that RTT is a monogenic disorder caused by mutations that alter the functionality of the methyl-CpG-binding domain (MBD) and the NCoR/SMRT interaction domain (NID) in *MECP2* [14,15,16]. This may simplify the complication of developing a treatment strategy. But, elucidation on the overlapped symptoms between those three proteins comprehensively on the molecular basis also seems necessary as the study about it remains scarce and it may provide meaningful insight, particularly for RTT. 

The MeCP2 structure has been determined using various experimental methods, while the structure of FOXG1 has only been investigated by predictions [17,18]. In the case of CDKL5, the structure of the amino-terminal kinase domain has already been identified, but that of the long carboxy-terminal tail has not been clarified [19]. These proteins have been suggested to contain polypeptide segments that are unable to fold spontaneously into three-dimensional structures; the so-called intrinsically disordered regions (IDRs) exist as dynamic ensembles that rapidly interconvert from molten globule (collapsed) to coiled or pre-molten globule (extended) as a result of the relatively flat energy landscapes [20,21]. The different entities of IDRs and ordered regions (displaying tertiary structures in native conditions) are dictated by the amino acid sequence; the former generally lack bulky hydrophobic residues [22]. Proteins are composed of either fully structured or fully disordered regions (with the latter referred to as intrinsically disordered proteins (IDPs) or a combination of the two, which is the case for most eukaryotic proteins [23]. Although protein function has traditionally been elucidated based on a well-defined structure, it is now widely acknowledged that IDRs contribute to diverse functions, which can be classified into six types: entropic chain activity, display site, chaperone, molecular effector, molecular assembler, and molecular scavenger [23,24,25,26]. Excluding entropic chain activity, IDRs adopt specific tertiary conformations—at least locally—in order to perform those functions by binding to other proteins, nucleic acids, membranes, and small molecules or responding to changes in their environment [20,27]. Hence, IDR structure varies over time—i.e., it exhibits spatiotemporal heterogeneity. Moreover, long IDRs contain more modification sites than fully ordered regions, and their flexibility provides more opportunities for displaying these sites [28,29]. These features explain how proteins with IDRs or IDPs interact with and are tightly regulated by various factors to ensure that appropriate levels of proteins are available at the right time to minimize the possibility of inappropriate protein–protein interactions [26]. Thus, misfolding and altered availability of proteins with IDRs or IDPs are more likely to be associated with disease states. Given a similarity in those properties, we proposed that a study concerning the link between MeCP2, CDKL5, and FOXG1 disordered structure properties with RTT or RTT-like syndrome collectively is necessary.

Restoring *Mecp2* gene function in an animal model abolished the symptoms of RTT. Growth factor stimulation (e.g., insulin-like growth factor 1) and the activation of neurotransmitter pathways (e.g., β2-adrenergic receptor pathway) can also partially rescue phenotypes of *Mecp2* knockout mice (RTT model mice), suggesting that the disorder is treatable [15,30,31]. In addition to gene therapy, reactivation of an inactivated X chromosome is known to be a new therapeutic method [32,33]. The therapeutic strategies of RTT are under development, and elucidation on this enigmatic disorder needs various points of view to make advances in understanding. Even though RTT has been determined as a monogenic disorder, the complex biological system compels us to necessarily broaden our perspective; moreover, MeCP2 contains an extensive amount of disordered regions which may facilitate binding with multiple partners. Considering several points above, we investigated the evolution and molecular features of MeCP2, CDKL5, and FOXG1 and their binding partners using phylogenetic profiling to gain a better understanding of their similarities. Additionally, we predicted the structural order–disorder propensity and assessed the evolutionary rates per site of MeCP2, CDKL5, and FOXG1 to investigate the relationships between disordered structure and other related properties with RTT.

## 2. Results

### 2.1. Structural Order–Disorder Properties of RTT and RTT-like Causing Proteins during Chordate Evolution

**Figure 1:**
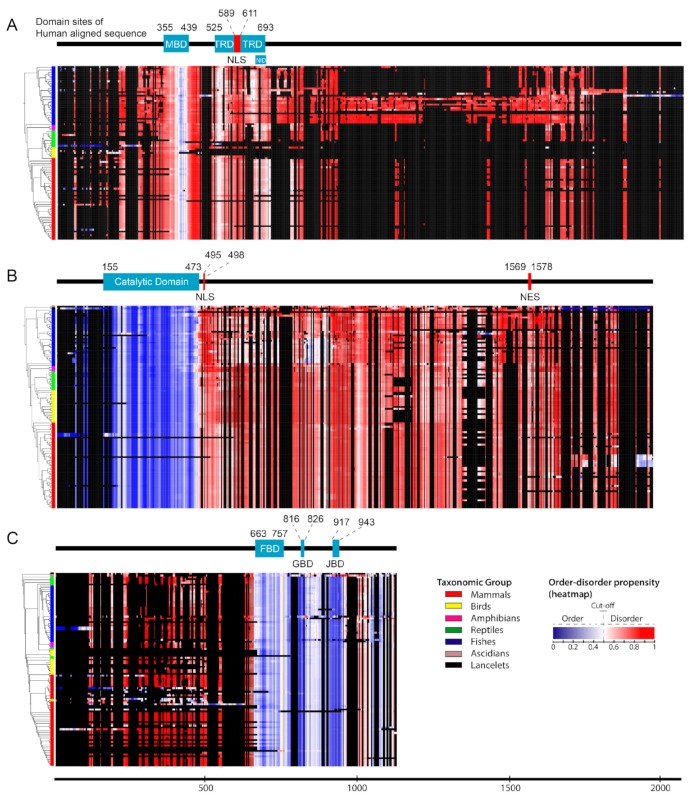
The order–disorder propensity of RTT and RTT-like causing proteins in chordates. Heat maps of the order–disorder propensity were generated according to the taxonomic positions in the phylogenetic tree (rows) and multiple sequence alignment (columns). The heat maps show a color gradient of blue (ordered) to red (disordered), with white as the boundary between the two and black as gaps. Colored boxes between the trees and heat maps indicate the taxonomic group, and bars above the heat maps indicate domain position in the multiple sequence alignment, with light blue and black areas indicating the domain and absence of a domain, respectively. (**A**–**C**) Heat maps for MeCP2 (**A**), CDKL5 (**B**), and FOXG1 (**C**) are shown. MBD, TRD, NID, FBD, GBD, JBD, NLS, and NES indicate methyl-CpG-binding domain, transcriptional repression domain, NCoR/SMRT interaction domain, forkhead binding domain, Groucho-binding domain, JARID1B binding domain, nuclear localization signal, and nuclear export signal, respectively.

We retrieved 97, 113, and 108 chordates sequences of MeCP2, CDKL5, and FOXG1, respectively, and constructed a heat map of the structural order–disorder propensity for each protein of these genes according to aligned sequences and taxonomic position in the phylogenetic tree (Supplementary Table S1 and Figure 1). This analysis was conducted in order to investigate the evolutionary patterns of structural properties. The results showed that all proteins harbored both ordered and disordered regions; by comparing their distribution to domain and non-domain regions, we found that the catalytic domain and non-domain regions of CDKL5 were ordered and disordered, respectively (Figure 1B). While most regions of MeCP2 were predicted to be disordered, some ordered structures were observed in the MBD (Figure 1A). Furthermore, FOXG1 showed a varied distribution of ordered–disordered regions corresponding to domain and non-domain regions, with the former predicted to be fully ordered (Figure 1C). Although insertions and deletions were frequently detected in disordered regions, particularly in MeCP2 and FOXG1 (Figure 1A,C), the structural order–disorder of all proteins showed to be stable in chordates, excluding a few conformational transitions of FOXG1 and CDKL5 in mammals and fishes, respectively. This indicated that the disordered regions of MeCP2, CDKL5, and FOXG1 tend to be functional either as an entropic chain, transient binding site, or permanent binding site in chordates. Additionally, insertions and deletions were frequently detected in disordered regions. This is caused by their flexibility, which makes sequence alignment difficult; a tendency of linear motifs to lie among the flexible disordered regions; and the permutation of functional modules with respect to others during evolution that is possible in disordered regions, such as SUMO modification sites in *Drosophila melanogaster* and human p53 that are located before and after the oligomerization domain, respectively [26,34].

### 2.2. Rate of Evolution per Site in RTT and RTT-like Causing Proteins

**Figure 2:**
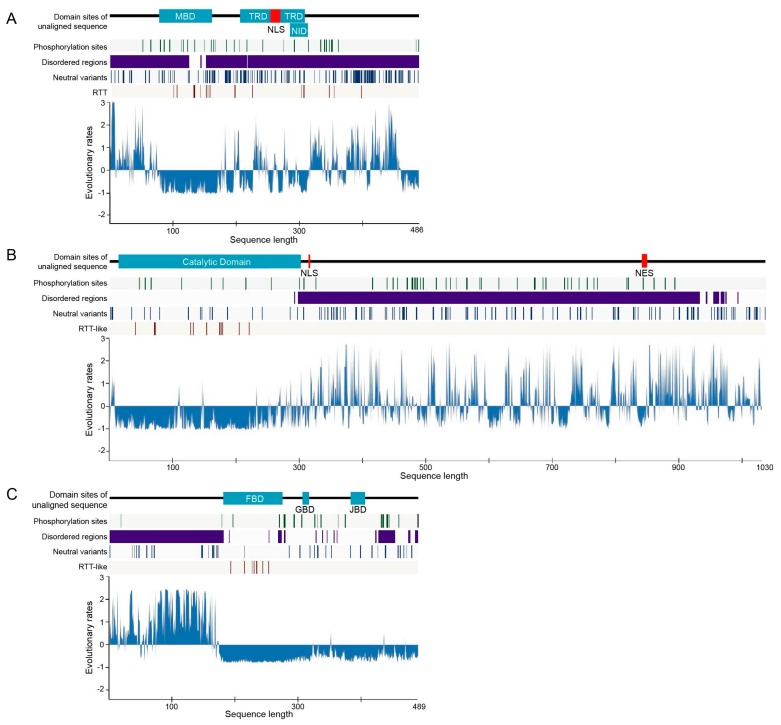
Rate of evolution per site in human RTT-related proteins. (**A**–**C**) Rates of amino acid substitution in MeCP2 (**A**), CDKL5 (**B**), and FOXG1 (**C**) are shown as blue areas. The bars above charts indicate the position of the domain in the human sequence, with light blue areas indicating the domain and black lines indicating no domain. Conserved phosphorylation sites, disordered region, single nucleotide polymorphisms in the general population, and pathogenic missense point mutation are plotted in green, purple, blue, and red lines, respectively. The *x* and *y* axes represent the sequence length and Z score of the evolutionary rates, respectively.

We calculated the evolutionary rates of MeCP2, CDKL5, and FOXG1 in chordates to investigate their relationships with structural features and the distribution of missense point mutations that have previously been suggested to contribute to RTT or RTT-like syndrome. We used the human sequence as a reference and determined standardized evolutionary rate scores (Z scores), with values greater than or less than zero reflecting evolution at a faster and slower than average rate, respectively (Figure 2 and Supplementary Table S2). Evolutionary rates per site showed similar patterns in all proteins, with low rates of evolution more commonly observed in domains and ordered regions; some exceptional cases such as the transcriptional repression domain (TRD) of MeCP2 showed a partial higher rate of amino acid substitution. On the other hand, non-domain regions that were also usually disordered—excluding the ordered region surrounding a domain in FOXG1—typically exhibited a higher evolutionary rate, although some regions with low rates of evolution were nonetheless detected (Figure 2). This was corroborated by the distribution of evolutionary rates for predicted structural order–disorder residues in the three proteins, with disordered residues showing a wide and overlapping distribution that reflected their conservation. The evolutionary rates of ordered and disordered regions are significantly distinct in those three proteins (*p* < 2.2e−16 for CDKL5 and FOXG1 and *p* < 6.409e−08 for MeCP2, Mann–Whitney U-test; Figure S1).

We identified structurally conserved disordered regions, with slowly and rapidly evolving residues reflecting constrained disorder and flexible disorder, respectively [26]. The flexible disorder has a constrained disordered structure despite having rapid evolution of residues; the amino acid substitutions of this property are constrained to residues that confer structural flexibility as the change from structurally disordered to ordered can affect protein function. This type of IDR typically functions as an entropic spring, flexible linker, or spacer without becoming structured and is frequently located outside the domain region [26,35,36,37]. In contrast, constrained disorder is associated with protein–protein interaction interfaces that adopt a structured conformation or undergo folding upon binding and are thus constrained in terms of sequence, while still requiring flexibility. This module can be present as short linear motifs (SLiMs) or intrinsically disordered domains (IDDs) [26,38]. These regions commonly have secondary structures that may be important for binding and, hence, slowing their evolutionary rates [36,39]. IDDs were observed in the MBD—which was predicted to be partly disordered—and in the TRD and NID of MeCP2; it is in accordance with previous reports that structured regions are found only in the MBD, while other regions are extensively disordered [17,18,40]. Most domains with conserved disordered regions are involved in DNA, RNA, and protein binding, which has been demonstrated by those domains of MeCP2 [41]. SLiMs are frequently located outside the domain and may display modification site. In this study, we predicted the constrained disorder regions and conserved phosphorylation sites located outside the domain to be associated with SLiMs, such as the region that spans after the catalytic domain to the C-terminus of human CDKL5.

### 2.3. Post-Translational Modifications (PTMs)

Phosphorylation is important for modulating the balance of proteins between the bound and unbound states, and previous studies reported that kinases target disordered proteins as many as twice, on average, the number of times they target structured proteins [42,43]. In this study, we predicted PTM (phosphorylation) sites in chordate sequences of MeCP2, CDKL5, and FOXG1 and predicted the conserved human phosphorylation sites to chordates in order to investigate the dynamics of their phosphorylation-related function. We found numerous conserved phosphorylation sites including 60/82 in CDKL5, 30/45 in MeCP2, and all 23 sites in FOXG1 in human (Figure 2 green lines and Supplementary Table S3). Most predicted human phosphorylation sites in MeCP2, CDKL5, and FOXG1 are conserved across chordates and are located in disordered regions; one exception is FOXG1, in which almost half of the phosphorylation sites are located in predicted ordered regions; structural disorder makes such sites accessible for phosphorylation. As PTMs affect the stability, turnover, interaction potential, and localization of proteins within the cell, proteins with disordered regions are more likely to be multifunctional [26]; accordingly, it has shown that MeCP2, CDKL5, and FOXG1 play multiple roles in the molecular basis.

### 2.4. Disease-Associated Missense Mutation Distribution in the Sequence of RTT and RTT-like Causing Proteins

Plotting missense mutations associated with diseases may yield crucial information on structure–function relationships and the features of the protein. We investigated missense mutations in human MeCP2, CDKL5, and FOXG1 that were previously associated with pathogenic RTT from RettBASE and examined the features of the associated sequences. There were 7, 12, and 18 individual amino acid sites in FOXG1, CDKL5, and MeCP2, respectively, that harbored pathogenic missense mutations associated or previously suggested to be associated with pathogenic RTT (Figure 2 and Supplementary Table S4). When the frequencies were combined with those of cases observed for each mutation, MeCP2 had a higher number of cases (1225) than CDKL5 (30) and FOXG1 (8) (Supplementary Tables S4 and S9). Pathogenic RTT or RTT-like-associated missense mutations were more frequently detected in domain regions for all proteins, and in ordered and slowly evolving regions for MeCP2 and CDKL5 (Supplementary Table S9). On the other hand, many mutation sites in MeCP2 were located close to (or in the case of Ser346Arg and Ser134Cys, overlapped with) phosphorylation sites (Figure 2), although the frequency of cases harboring these mutation sites was low (only one for each).

### 2.5. Phylogenetic Profiling of RTT and RTT-like Causing Proteins and Their Interaction Partners

**Figure 3:**
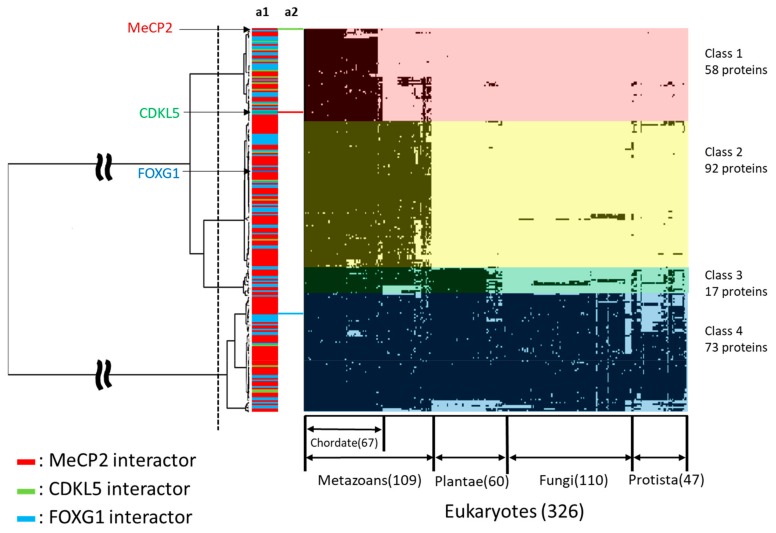
Phylogenetic profiling of MeCP2, CDKL5, and FOXG1 proteins and their interaction partners. The horizontal axis shows 326 eukaryotes for which whole genome sequences are available, and the vertical axis shows 240 human proteins related to RTT. Bar in a1 and a2 shows MeCP2-interactor (red), CDKL5-interactor (green), FOXG1-interactor (blue), respectively. The human orthologous proteins in each species are shown in black. The phylogenetic tree was divided into four clusters (Class 1–4); those conserved across chordates, metazoan, multicellular, and eukaryotes are shown.

We retrieved 240 human proteins interacting with MeCP2, CDKL5, and FOXG1 from BioGRID and UniProt databases (Supplementary Table S5) [44,45]. To illuminate the interconnection of MeCP2, CDKL5, and FOXG1 binding partners as well as their evolutionary relationship, we conducted phylogenetic profiling and cluster analysis of 326 eukaryotes using the retrieved sequences and the sequences of the three proteins, MeCP2, CDKL5, and FOXG1, as queries (Figure 3, Supplementary Table S6). The results showed that the dataset was divided into four clusters, which were defined as Classes 1 to 4. There were 58 conserved proteins in chordates of Class 1, 92 in metazoans of Class 2, 17 in multicellular of Class 3, and 73 in eukaryotes of Class 4. MeCP2 and CDKL5 belonged to Class 1, whereas FOXG1 belonged to Class 2 (Figure 3). FOXG1 and MECP2 showed to have many binding partners that act as a transcription factor or gene expression regulator. In contrast, CDKL5 tend to bind to a fewer number of proteins having functions in regulating cell adhesion, ciliogenesis, and cell proliferation; however, this protein has been shown to interact with MeCP2. As RTT has been determined to occur from the altered functionality of MBD and NID of MECP2, we focused on the widely known binding partners of these domains, such as SIN3 transcription regulator family member A (SIN3A), histone deacetylase (HDAC)1, and nuclear receptor corepressor (NCOR) which play roles as co-repressor complexes. Even though FOXG1 does not directly bind to MeCP2, we found that the binding partners of MeCP2 co-repressor complex are also associated with FOXG1 binding partners that also act as co-repressor complexes such as special AT-rich sequence-binding protein (SATB)2, lysine-specific histone demethylase (KDM)1A, SWI/SNF-related matrix-associated actin-dependent regulator of chromatin subfamily (SMARC)A member 5, A-kinase anchor protein (AKAP)8, of which are ancient proteins within Classes 3 and 4.

### 2.6. Subcellular Localization and Gene Ontology (GO) Analysis

**Figure 4:**
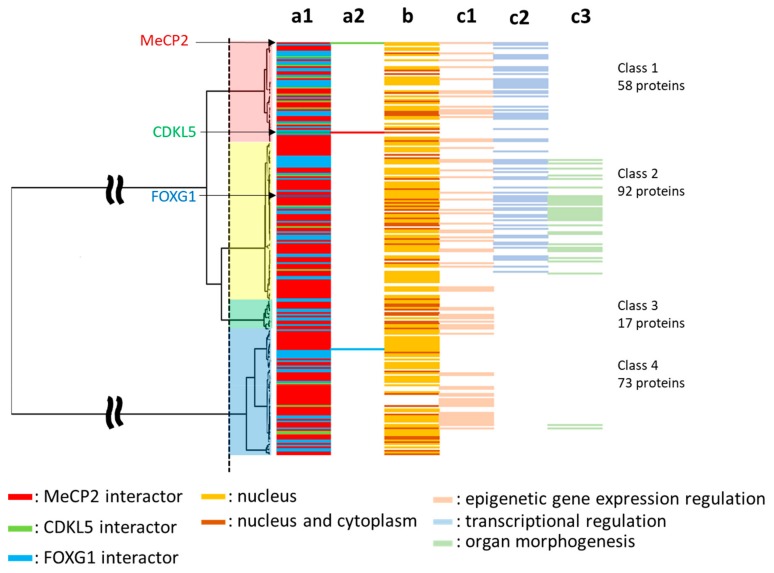
Subcellular localization and specific GO categories of human RTT-related proteins: Phylogenetic trees show interactors, subcellular localization, and specific GO categories for each protein. The vertical axis shows 240 RTT-related proteins, and each bar shows MeCP2-interactor (red), CDKL5-interactor (green), and FOXG1-interactor (blue) (a1 and a2); cellular localization (b); epigenetic regulation of gene expression (c1); transcriptional regulation (c2); and organogenesis (c3).

We predicted the subcellular localization of each protein and GO categories in each class for the evolutionary classification (Figure 4, Supplementary Table S7). Specific GO categories included epigenetic regulation of gene expression, transcriptional regulation, and organogenesis or organ morphogenesis (Figure 4). We confirmed the evolutionary trends of proteins with specific GO categories and their subcellular localization and found that 129 and 48 proteins in Classes 1–4 were expressed in the nucleus only or the nucleus and cytoplasm, respectively. Proteins in Classes 1–4 were represented in the epigenetic regulation of gene expression category, whereas transcriptional regulation was observed only in Classes 1 and 2, and organogenesis and organ morphogenesis were mainly observed in Class 2 (Figure 4).

### 2.7. Tissue and Organ Localization

Tissue and organ expression data for 237 proteins were extracted from The Human Protein Atlas as transcripts per million (TPM) values [46]. In addition, four proteins were not expressed in the cerebral cortex. Tissues and organs with specific expression were identified using 195 RTT-related human proteins as queries (Figure S2, Supplementary Table S8). There were nine proteins that were specifically expressed in the cerebral cortex including apolipoprotein E, CDKL5, SATB2, spalt-like transcription factor (SALL)1, zinc finger protein (ZNF)483, FOXG1, (sex-determining region Y)-box (SOX)2, homeodomain-interacting protein kinase (HIPK)2, and histone cluster 2 H3 family member A.

## 3. Discussion

RTT is a progressive postnatal neurodevelopmental disorder; three individual genes, *MECP2*, *CDKL5*, and *FOXG1*, have previously been thought to be the cause of its variants with the altered *MECP2* as the major contributor. Later, it was suggested that RTT is a monogenic disorder caused by either null mutations or mutations that alter the MBD or NID functions of *MECP2* [15,16,47]. MBD and NID facilitate the binding of MeCP2 to modified cytosine in chromatin and recruitment of the NCOR-SMRT complex, respectively; their combination is vital for MeCP2’s role as a repressor [48,49]. The altered forms in the other two genes which were previously characterized as variants of RTT were designed as distinct disorders with several overlapping symptoms to RTT. The three proteins have similar extensive amount of disordered regions and play important roles in the brain. The disordered structure itself is a unique property in protein that may contribute to the interaction with a diverse binding partner and the versatility of a protein. While the three proteins may show similar symptoms in the altered form, the investigation on their similarity in the molecular basis remains scarce, particularly on the disordered structure properties and their binding partners. Focusing on RTT, we investigated the evolution of their disordered structures and their binding partners through prediction and phylogenetic profiling, respectively. This approach is important to give an insight into the similarity of biological systems of those proteins structurally and evolutionarily, which may provide useful information for the development of a RTT therapy strategy. RTT itself has attracted considerable attention as its causative protein displays features related to epigenetics and have been shown to have partially or fully disordered structures.

All three proteins have been experimentally determined to play roles and are abundant in the brain, especially the MeCP2_e1 and hCDKL5_1 isoforms [50,51]. It is confirmed by the emergence of neurological impairments in the altered availability or forms of either protein. Through evolutionary analysis and IDRs properties, we provide an additional point of view for that feature. Phylogenetic profiling analysis of MeCP2, CDKL5, and FOXG1 and their interacting proteins showed that 240 molecules formed four clusters—i.e., chordates, metazoans, multicellular, and eukaryotes. Among the three, only *FOXG1* was a member of Class 2, which comprises genes acquired during metazoan evolution, whereas the acquisition of *MECP2* and *CDKL5* was correlated with chordate evolution. The acquisition of *CDKL5* and *MECP2*, and *FOXG1* may contribute to the development of the chordate brain and metazoan nervous system during evolution, respectively. Additionally, order–disorder structure predictions revealed that all three proteins had order–disorder structures that were relatively conserved across chordates. Human MeCP2, CDKL5, and FOXG1 phosphorylation sites were also shown to be relatively conserved to chordates. IDRs properties provide proteins with more interaction areas and PTMs sites, spatiotemporal heterogeneity of structure, and ability to associate and dissociate easily with binding partners. Hence, proteins with long IDRs are likely to have a capacity to bind to many different partners. Accordingly, all three proteins were shown to have multiple binding partners, and FOXG1 and MeCP2 displayed the highest number of partners, some of which were evolutionarily acquired before the metazoan evolved. By cooperating with various proteins partners, particularly the co-repressor complex, FOXG1 or MeCP2 can modulate the expression and suppression of different genes [15,52]. The co-repressor complex itself denotes a conserved mechanism that manifests in diverse forms and may have several functional entities depending on the context in which they are recruited [53]. This indicates the necessity to regulate either FOXG1 or MeCP2 concentration precisely; otherwise, altered availability is likely to be deleterious. Several studies have shown that either overexpression or under-expression of MeCP2 and FOXG1 corresponds to neurological deficits; this phenomenon may not independent from their co-repressor complex that has been showed to play roles in neurogenesis and neuron maturation for FOXG1, and MeCP2, respectively [7,15,52]. On the other hand, CDKL5 binds to a fewer number of proteins that have functions in regulating cell adhesion, ciliogenesis, and cell proliferation. We hypothetically suggest that the amount of CDKL5 binding partners is underestimated since this protein was predicted to have relatively long disordered regions with many constrained disorder features and phosphorylation sites; it also has fewer insertions and deletions than either MeCP2 or FOXG1 along the evolution. 

FOXG1 is a transcriptional factor playing an essential role in ventral telencephalon development; it serves as a hallmark of the telencephalon in vertebrates [52,54]. Among the 237 Class 1 or 2 genes, 233 were detected in the cerebral cortex, with nine expressed at a high level (Figure S2). Seven genes were acquired during metazoan evolution, of which four and three encode MeCP2- and FOXG1-interacting molecules, respectively. Since FOXG1 was also acquired during metazoan evolution, acquisition of FOXG1, SATB2, and SALL1 may have played essential roles in development of the neocortex. FOXG1 is transiently expressed in neuronal progenitor cells and regulates their migration to the cortical plate [55]. During this process, FOXG1 expression is upregulated, which contributes to cortical plate development [56]. Similarly, the FOXG1-interacting chromatin remodeling factor SATB2 was found to be expressed in the cortical plate and regulates neocortical development [54,55]. Therefore, it is conceivable that transcriptional co-operation between FOXG1 and SATB2 mediates the laminarization of the neocortex. In support of this possibility, patients with the SATB2 mutation exhibit an RTT-like phenotype [57,58]. There is no direct interplay reported for MeCP2 and FOXG1. The causative regions in the altered form of these proteins that result in the development of RTT or RTT-like disorder exhibited similar functions in regulating the other genes’ expression, but likely via a distinct pathway. We suggest that FOXG1 is not a potential target for developing treatment for RTT. However, induced pluripotent stem cell (iPSC)-derived neurons generated from FOXG1+/− patients and patients with MECP2 and CDKL5 mutations reportedly exhibited a similar increase in synaptic cell adhesion protein orphan glutamate receptor δ-1 subunit (GluD1) expression; this result indicates the need for further study to reveal the mechanism of each protein and might be implicated in the clinical symptom overlap among FOXG1-, CDKL5- and MECP2-related syndromes [52,59,60].

CDKL5 belongs to the same molecular pathway of MeCP2. MeCP2 was acquired during chordate evolution; a prerequisite for this step was the acquisition of MeCP2-interacting molecules such as ZNF483, SOX2, HIPK2, and HIST2H2A. The MeCP2 kinase HIPK2 was shown to be required for the induction of apoptotic cell death in neuronal and other cell types via phosphorylation of the MeCP2 N-terminus [61]. Given that CDKL5, another MeCP2 kinase was also acquired during chordate evolution; it is possible that HIPK2 and CDKL5 cooperate to activate MeCP2 during neocortical development. Since apoptotic cell death increased in *Cdkl5* knockout mouse brain, CDKL5 probably has a suppressive function in the apoptosis process in contrast to HIPK2 [62]. Therefore, functional division of their kinases through phosphorylation of MeCP2 is an important issue. Indeed, the CDKL5-interacting domain was shown to be associated with the C-terminus of MeCP2 [63]. Hence, CDKL5 may phosphorylate the carboxy terminus. Thus, both HIPK2 and CDKL5 may activate MeCP2 by phosphorylating different regions of the protein. It has been suggested that MeCP2 also suppresses CDKL5 transcription and that CDKL5 overexpression may also contribute to the typical RTT symptoms [64]. Hence, aiming the catalytic domain of CDKL5 as the key target for developing alternative strategies to treat classical RTT may be essential since its sole impairment resulted in some symptoms that overlapped with those of classical RTT. Additionally, the CDKL5 disordered region, which spans after the catalytic domain to the C-terminus, is suggested to have many SLiMs. The linear motifs theoretically help to determine the various fates of a protein including subcellular localization, stability, and degradation; these motifs are also able to promote recruitment of binding factors and facilitating post-translational modifications [26,38]. Since these motifs typically regulate low-affinity interactions, they can bind to molecules with different structures of similar affinity and facilitate transient-binding, which are favorable properties for drug targets. Accordingly, this region appears to be a potential target for classical RTT treatment. However, this should also consider the expression levels of CDKL5 which are highly modulated spatiotemporally [64,65].

IDRs show unique properties within protein which challenges the traditional viewpoint of the protein structure paradigm. They have differences in residue composition, intramolecular contacts, and functions to ordered regions which cause different evolutionary rates. Generally, they evolve more rapidly than ordered regions, owing to the different accepted point mutations. However, some disordered regions can be highly constrained as they may play crucial roles and have multiple functions; assessing the evolutionary rate of IDRs may thus reveal crucial protein-specific amino acids in the biological system [66]. In this study, we found a unique relationship between evolutionary rates of disordered regions and symptoms of a disease caused by FOXG1. The N-terminus residues of FOXG1 are highly variable and constrained to be disordered, while the residues from FBD to the C-terminus are constrained and contain an ordered structure. It has been reported that mutations in the N-terminal are more likely to be associated with severe phenotypes, and mutations in the C-terminal are associated with milder phenotypes [52]. We reported and predicted a phosphorylation site located in Ser 19 to be conserved in chordates even though it is located among flexible disordered regions; casein kinase 1 (CK1) modifies this site and promotes the nuclear import of FOXG1, which corresponds to neurogenesis in the forebrain [67]. This explains that a flexible disordered region can retain its functional module from phosphorylation, despite harboring numerous insertions and deletions, and that severe phenotypes may result from the altered function of Ser 19 of FOXG1. 

Among 236 male testis expressing RTT-related genes, 47 genes expressed at a high level. Because paternal-derived de novo mutation has been shown to affect X-linked MeCP2-related female Rett syndrome [6,68], paternally expressing mutation in these genes may affect the sperm-derived genetic and/or epigenetic inheritance that influence the cause of Rett syndrome in a daughter. Further studies are required to analyze these possibilities.

It is important to remember that the features of structural order–disorder and phosphorylation sites in this study have been inferred using linear sequence predictors and that the sequences and mutation points were retrieved from databases whose data have been collected from studies with various methods. It should be considered that we use canonical isoforms instead of predominant brain isoforms, this option may be able to be applied computationally but should be of concern experimentally. This study provides suggestive or hypothetical conclusions, thus further experimental study is important to verify the findings of this study. Ultimately, the results can still be used and considered as a basis for further identification.

## 4. Materials and Methods

### 4.1. Sequence Retrieval, Alignment, and Phylogenetic Analysis of MeCP2, CDKL5, and FOXG1 Proteins

Orthologous sequences of human RTT and RTT-like causing proteins (MeCP2, CDKL5, and FOXG1) in chordates were retrieved from the Kyoto Encyclopedia of Genes and Genomes (KEGG) sequence similarity database (https://www.kegg.jp/kegg/ssdb/) with a Smith–Waterman similarity score threshold of 100 and the bidirectional best hits (best–best hits) option [69]. We primarily used the canonical isoforms MeCP2_e2 and hCDKL_5 instead of those the predominant isoforms in the human brain, MeCP2_e1, and hCDKL5_1. MeCP2_e2 is the most characterized isoform relative to MeCP2_e1, and RettBASE has chosen to name the variants MeCP2_e2 due to historical reason. Variants specific to MeCP2_e1 are still reported in RettBASE with the prefix MeCP2_e1 in the database, but we decided to exclude them in our analysis as we only found one variant that meets our criteria and it cannot be included within the MeCP2_e2 sequence as they differ in the *N*-terminal region; however, we still reported that variant in our Supplementary Data. CDKL5 has a similar case as MeCP2, but the differences of sequences between hCDKL_5 and hCDKL5_1 are located in the C-terminal region (905–1030 a.a) which does not shift the reported Rett-like variants in the catalytic domain. We selected this option as we primarily collected the RTT and RTT-like variants from RettBASE. The used isoforms do not differ greatly to those predominant brain isoforms. The highest similarity score for each species was used for each of those proteins to minimize redundancy. Datasets were created for each protein and then aligned using MAFFT v.7 (https://mafft.cbrc.jp/alignment/software/) with the iterative refinement method (FFT-NS-i), with a maximum of 1000 iterations [70]. Phylogenetic trees were constructed with the maximum likelihood method using RAxML-HPC2 BlackBox with the RAxML automatic bootstrapping option using Jones, Taylor, and Thornton amino acid substitutions with the + F method and gamma shape parameter (JTT + F + G) model for MeCP2 and CDKL5, and the JTT + G model for FOXG1, which were selected as the best fit models under the Bayesian information criterion (BIC) by ModelTest-NG [71,72]. The outgroup for each tree was selected based on the NCBI Taxonomy Common Tree for the common ancestor within the dataset [73]. Reconstruction of phylogenetic trees and calculation of models were performed in CIPRES Science Gateway (http://www.phylo.org/) [74].

### 4.2. Structural Order–Disorder Prediction and Secondary Structure Predictions

The structural order–disorder propensity of each protein was predicted using IUPred2A (https://iupred2a.elte.hu/) [75] using the option for long disordered regions. This prediction had values ranging from 0 (strong propensity for an ordered structure) to 1 (strong propensity for a disordered structure), with 0.5 as the cut-off between the propensity for order and disorder. The results for each site of each protein were mapped onto its sequence alignment and taxon position in the phylogenetic tree using iTOL (https://itol.embl.de/) [76].

### 4.3. Rate of Evolution per Site

We calculated the rate of evolution per site of human CDKL5, FOXG1, and MeCP2 relative to their orthologs using Rate4site (https://m.tau.ac.il/~itaymay/cp/rate4site.html) [77]. The aligned sequences of each protein dataset were calculated using the empirical Bayesian principle with the JTT model and 16 discrete categories of the prior gamma distribution. Gaps were treated as missing data, and outputs were standardized as Z scores. The results of the rate of evolution of each residue were then integrated with the structural order–disorder prediction result, and the distribution of the rate of evolution in the structural order and disorder of each protein was evaluated with the Mann–Whitney U-test using R software.

### 4.4. PTM Prediction

We predicted phosphorylation sites using NetPhos 3.1 (http://www.cbs.dtu.dk/services/NetPhos/) [78] to infer PTM sites conserved between human CDKL5, FOXG1, and MeCP2 sequences and their orthologs. The predictions had values ranging from 0 (strong propensity for obtaining a negative result) to 1 (strong propensity for obtaining a positive result); we used 0.75 as a cut-off to divide the negative and positive results. The prediction results for each sequence were plotted following multiple sequence alignment of each protein dataset. Predicted PTM sites in each dataset were considered as conserved through evolution if they had a positive value according to the 50% majority rule of the amount of sequence in the alignment.

### 4.5. Point Mutations in MeCP2, CDKL5, and FOXG1

Point mutations in CDKL5, FOXG1, and MeCP2 were identified from RettBASE (http://mecp2.chw.edu.au/) [79]. The amount of mutations variants in general in RettBASE are 929, 298, and 44 for MeCP2, CDKL5, and FOXG1, respectively. We only selected missense mutations that were associated with pathogenic RTT. Additionally, non-pathogenic polymorphisms in the general population for comparison were extracted from the Exome Aggregation Consortium database (http://exac.broadinstitute.org) [80].

### 4.6. Phylogenetic Profiling and Cluster Analyses of Human MeCP2, CDKL5, and FOXG1 and Their Interacting Proteins

Sequences of human MeCP2, CDKL5, and FOXG1 and their interaction partners identified with BioGRID (https://thebiogrid.org/; release 2019_03) were obtained from the UniProtKB/Swiss-Prot database (https://www.uniprot.org/help/uniprotkb; release 2019_04) and used as the dataset [45,46]. We generated phylogenetic profiles of 326 eukaryotes in the KEGG database (https://www.genome.jp/kegg/) using the dataset as a query [81]. Phylogenetic profiling is a method for detecting the presence or absence of orthologous proteins in a target organism [82]. The presence or absence of proteins homologous to the query in each species was determined using KEGG Ortholog Cluster (https://www.genome.jp/tools/oc/; release 2019_04), this tool uses Smith–Waterman similarity scores of ≥150 and symmetric similarity measures to classify the ortholog genes [83]. We suggest that it is a reliable tool to get ortholog data. Profiles were determined based on the Manhattan distance and then clustered using Ward’s method [84].

### 4.7. Protein Expression in Human Tissues

Expression levels of human RTT-related proteins in each tissue were extracted from the Human Protein Atlas (https://www.proteinatlas.org/; release 2019_4) [45] and classified into 37 tissues. The protein expression level was determined using the TPM value, which was corrected for protein expression by gene length. Comparisons of protein expression levels were not shown as a ratio so that proteins with high expression did not skew the results (Equations (1)–(3)). The mean and standard deviation were derived from Equations (1) and (2), and the range was obtained from Equation (3). The range in Equation (3) was taken as the tissue for each of the specifically expressed proteins—i.e., the value was “1” when included in the range of Equation (3) and “0” when it was not included in the expression level of each protein expressed as a percentage. The procedure yielded human protein-specific expression profiles in the context of RTT.

(1) $$\mu =\;\frac{1}{n}\overset{n}{\underset{i=0}{\sum }}x_{i}$$

(2) $$s=\;\sqrt{\frac{1}{n}\overset{n}{\underset{i=0}{\sum }}{\left(x_{i}-\mu \right)}^{2}}$$

(3) μ+1.65×s <x

Here, *μ*, *s*, *n*, and *x* are the mean, standard deviation, number of samples, and one sample, respectively. The value of 1.65 in Equation (3) is the standard confidence factor for extracting data outside the 90% confidence interval.

### 4.8. GO Analysis

Specific GO categories in the target protein group were obtained using the Panther tool [85]. Categories with an appearance frequency of *p* < 0.05 were defined as protein group-specific. In this study, we obtained GO categories specific for human proteins related to RTT that were classified based on defined functions.

## 5. Conclusions

In the last two decades, effort on elucidating RTT has shown a promising trend towards developing a reliable treatment for this disorder. Given a similarity in IDR properties and several overlapping symptoms, we investigated the evolution of MeCP2, CDKL5, and FOXG1 disordered structures and their binding partners through prediction and phylogenetic profiling, respectively. Here, we provided insight to the structural characteristics, evolution and interaction landscapes of those three proteins related to RTT. We suggested that the disordered structures of MECP2, CDKL5, and FOXG1 contribute to the versatility in brain development and may play a crucial role in brain evolution in chordates. We hypothetically suggested that CDKL5 could be a potential target for RTT treatment, particularly by targeting its disordered structure that spans after the catalytic domain to the C-terminus, which shows abundant linear motifs that can bind to molecules with different structures of similar affinity. Finally, this study may provide valuable guidance for experimental research, particularly on the relationship between RTT and disordered regions.

## Supplementary Materials

Supplementary materials can be found at https://www.mdpi.com/1422-0067/20/22/5593/s1.

## Abbreviations

a.a	Amino acids
AKAP8	A-kinase anchor protein 8
APOE	Apolipoprotein E
BioGRID	Biological General Repository for Interaction Datasets
CDKL5	Cyclin-dependent kinase-like 5
CIPRES	Cyberinfrastructure for Phylogenetic Research
CK1	Casein kinase 1
DOI	Digital object identifier
FBD	Forkhead box domain
FOXG1	Forkhead box protein G1
GBD	Groucho-binding domain
GluD1	Glutamate dehydrogenase 1
GO	Gene Ontology
HDAC1	Histone deacetylase 1
HIPK2	Homeodomain-interacting protein kinase 2
IDDs	Intrinsically disordered domains
IDPs	Intrinsically disordered proteins
IDRs	Intrinsically disordered regions
iPSC	Induced pluripotent stem cell
iTOL	Interactive Tree of Life
IUPred	Prediction of Intrinsically Unstructured Proteins
JBD	JARID1B-binding domain
JARID1B	Histone Demethylase Jumonji AT-rich Interactive Domain
JTT	The Jones, Taylor, and Thornton
KDM1A	Lysine-specific histone demethylase 1A
KEGG	Kyoto Encyclopedia of Genes and Genomes
MAFFT	Modified Multiple Alignment Fast Fourier Transform
MBD	Methyl-CpG-binding domain
MeCP2	Methyl-CpG-binding protein 2
NCOR	Nuclear receptor corepressor
NCoR/SMRT	Nuclear receptor co-repressor/silencing mediator of retinoic acid and thyroid hormone receptor
NES	Nuclear export signal
NLS	Nuclear localization signal
NID	NCoR/SMRT interaction domain
OMIM	Online Mendelian Inheritance in Man
PTM	Post-translational modification
RAxML-HPC2	Randomized Axelerated Maximum Likelihood for High-Performance Computing 2
RTT	Rett syndrome
RettBASE	Rett syndrome Variation Database
SALL1	Spalt-like transcription factor 1
SATB2	Special AT-rich sequence-binding protein 2
SIN3A	SIN3 transcription regulator family member A
SLiMs	Short linear motifs
SMARCA5	SWI/SNF-related matrix-associated actin-dependent regulator of chromatin subfamily A member 5
SOX2	SRY-box transcription factor 2
SSDB	Sequence Similarity DataBase
TRD	Transcriptional repression domain
TPM	Transcripts per million
ZNF483	zinc finger protein (ZNF)483
