# Supplementary material for: In Silico Study of Rett Syndrome Treatment-Related Genes, MECP2, CDKL5, and FOXG1, by Evolutionary Classification and Disordered Region Assessment
Source: Int J Mol Sci. 2019 Nov 8;20(22):5593. doi: 10.3390/ijms20225593 (PMC6888432; doi:10.3390/ijms20225593)
Supplement: Supplementary file 1 [file ijms-20-05593-s001.zip › supplementary figure.docx]

In Silico Study of Rett Syndrome Treatment-Related Genes, *MECP2*, *CDKL5*, and *FOXG1,* by Evolutionary Classification and Disordered Region Assessment

Muhamad Fahmi ^1^, Gen Yasui ^1^, Kaito Seki ^1^, Syouichi Katayama ^2^, Takako Kaneko-Kawano ^2^, Tetsuya Inazu ^2^, Yukihiko Kubota ^3^ and Masahiro Ito ^1,3,^*

^1^ Advanced Life Sciences Program, Graduate School of Life Sciences, Ritsumeikan University, Kusatsu, Shiga 525-8577, Japan; [gr0343rp@ed.ritsumei.ac.jp](mailto:gr0343rp@ed.ritsumei.ac.jp) (M.F.), [sj0048hh@ed.ritsumei.ac.jp (G.Y.)](mailto:sj0048hh@ed.ritsumei.ac.jp(G.Y.)), [sj0036kf@ed.ritsumei.ac.jp (K.S.)](mailto:sj0036kf@ed.ritsumei.ac.jp(K.S.)), [yukubota@fc.ritsumei.ac.jp](mailto:yukubota@fc.ritsumei.ac.jp)(Y.K.), [maito@sk.ritsumei.ac.jp](mailto:maito@sk.ritsumei.ac.jp) (M.I.)

^2^ Department of Pharmacy, College of Pharmaceutical Sciences, Ritsumeikan University, Kusatsu, Shiga 525-8577, Japan; [s-kata@fc.ritsumei.ac.jp (S.K.)](mailto:s-kata@fc.ritsumei.ac.jp(S.K.)), [takanek@fc.ritsumei.ac.jp](mailto:takanek@fc.ritsumei.ac.jp) (T.K.-K.), [tinazu@fc.ritsumei.ac.jp](mailto:tinazu@fc.ritsumei.ac.jp) (T.I.)

^3^ Department of Bioinformatics, College of Life Sciences, Ritsumeikan University, Kusatsu, Shiga 525-8577, Japan

***** Correspondence: maito@sk.ritsumei.ac.jp (M.I.)


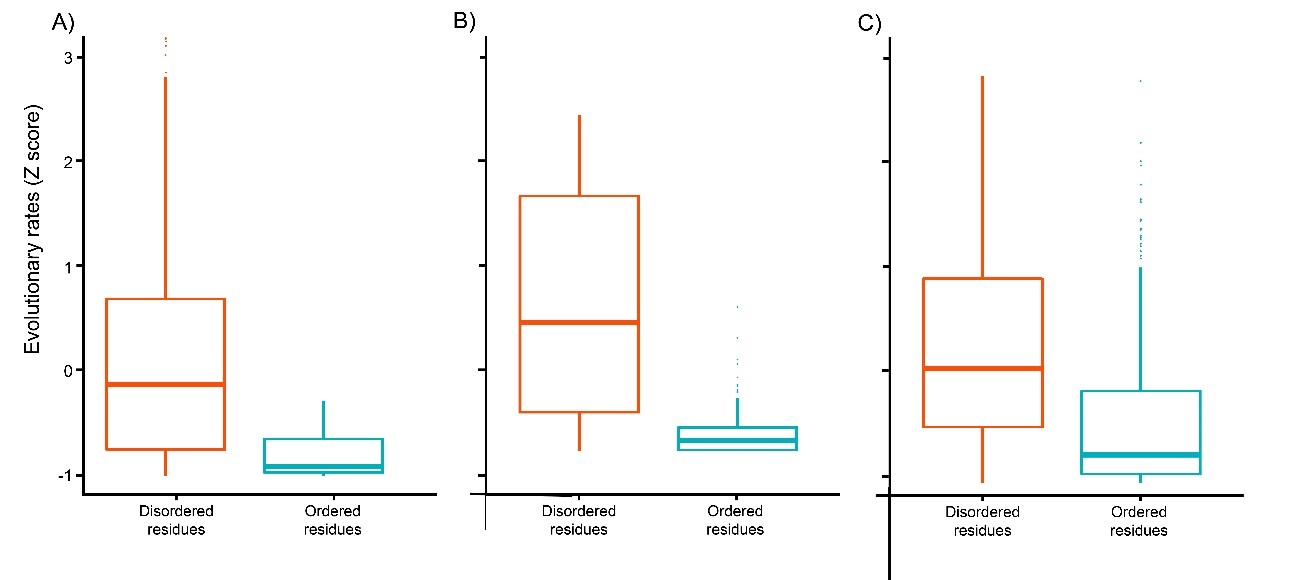


**Figure S1.** Boxplots of evolutionary rates for predicted structural order–disorder residues of human RTT and RTT-like causing proteins. (A–C) Boxes representing predicted ordered (blue) and disordered (red) structure residues in MECP2 (A), CDKL5 (B), and FOXG1 (C). The x and y axes represent predicted conformation and Z score of evolutionary rates, respectively.


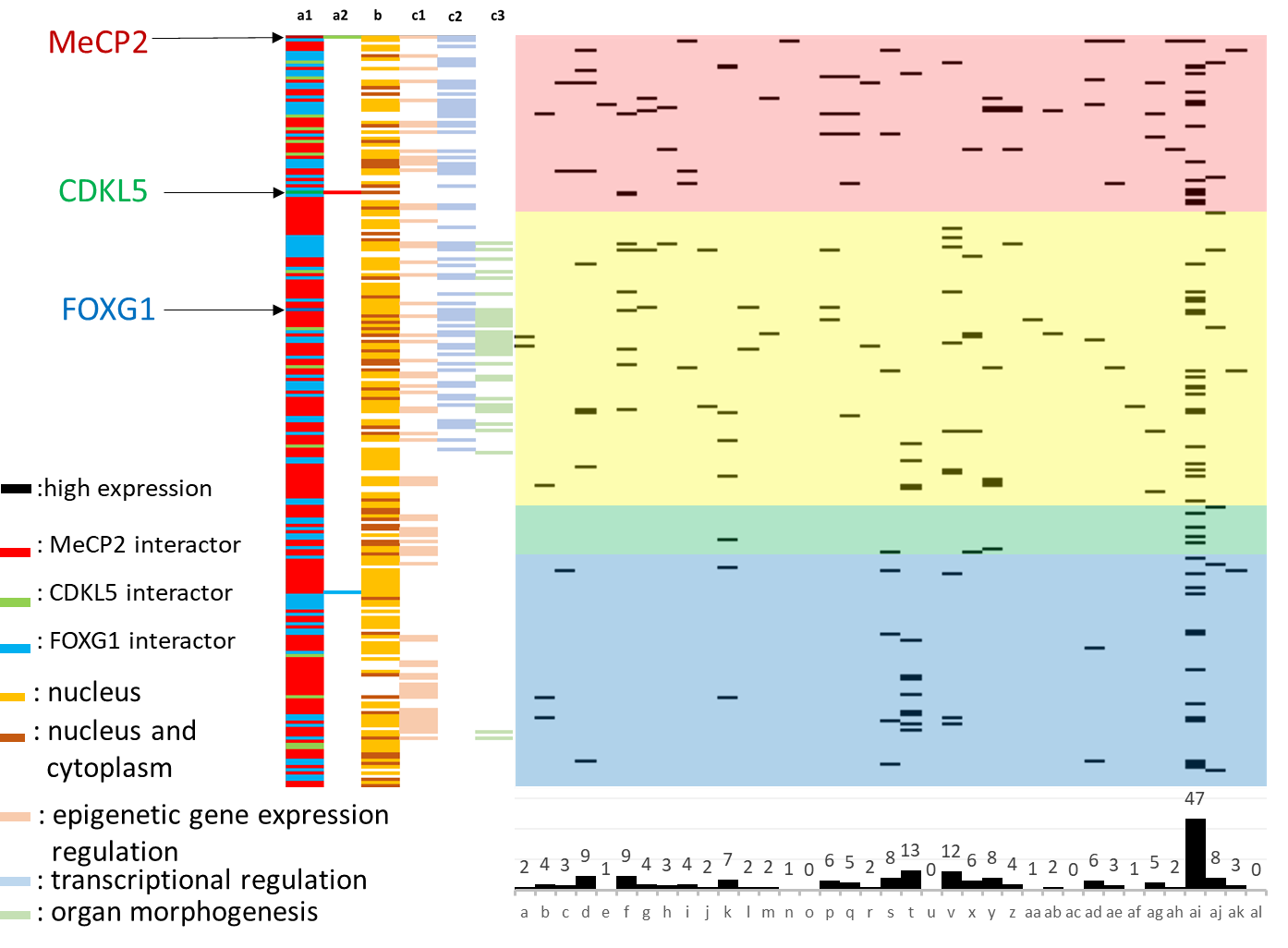


**Figure S2.** Tissue and organ expression analysis of human RTT-related proteins. The vertical axis shows 237 RTT-related proteins, and each bar shows MeCP2-interacter (red), CDKL5-interacter (green), and FOXG1-interactor (blue) (a1 and a2); cellular localization (b); epigenetic regulation of gene expression (c1); transcriptional regulation (c2); and organogenesis (c3). The horizontal axis shows 37 tissue types classified according to the Human Protein Atlas [37]. The tissue expressing each protein satisfying the range determined with Equation 3 is shown in black. The lower part of the figure shows the number of specifically expressed proteins. a, adipose tissue; b, adrenal gland; c, appendix; d, bone marrow; e, breast; f, cerebral cortex; g, cervix; uterine; h, colon; i, duodenum; j, endometrium; k, epididymis; l, esophagus; m, fallopian tube; n, gallbladder; o, heart muscle; p, kidney; q, liver; r, lung; s, lymph node; t, ovary; u, pancreas; v, parathyroid gland; x, placenta; y, prostate; z, rectum; aa, salivary gland; ab, seminal vesicle; ac, skeletal muscle; ad, skin; ae, small intestine; af, smooth muscle; ag, spleen; ah, stomach; ai, testis; aj, thyroid gland; ak, tonsil; al, urinary bladder.


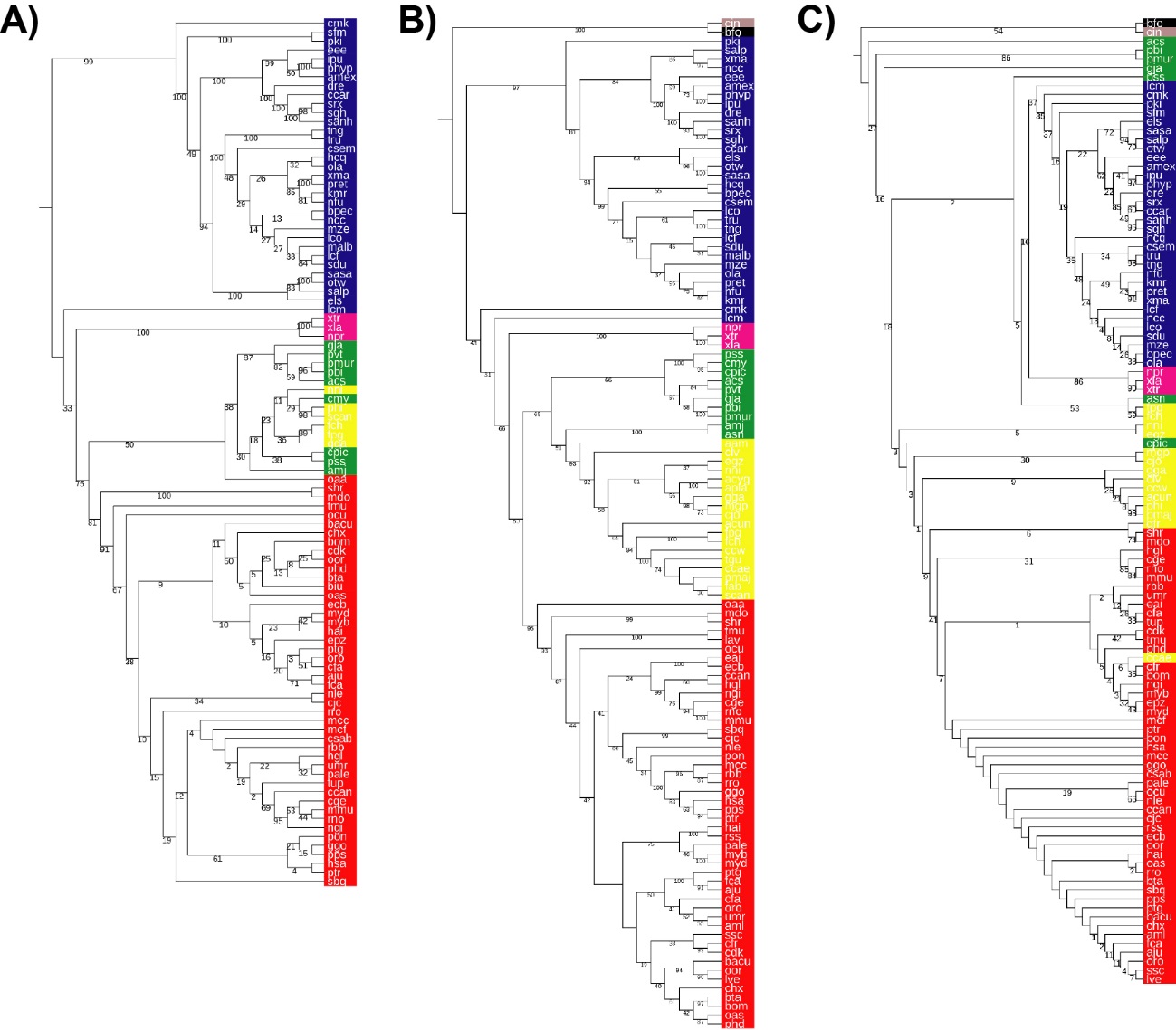


**Figure S3.** Phylogenetic trees of MECP2, CDKL5, and FOXG1 with maximum likelihood bootstrap. Color bars in the tips of trees indicate the taxa of species following to figure 1.
